# P-784. Early Prediction of Inducible Macrolide Resistance For *Mycobacterium abscessus* –Minimum Inhibitory Concentrations at Day 7 Versus Day 14

**DOI:** 10.1093/ofid/ofae631.978

**Published:** 2025-01-29

**Authors:** Liang-En Hwang, Aristine Cheng, Jung-Yien Chien

**Affiliations:** National Taiwan University Hospital, Banciao, New Taipei, Taiwan (Republic of China); National Taiwan University Hospital, Banciao, New Taipei, Taiwan (Republic of China); National Taiwan University Hospital, Banciao, New Taipei, Taiwan (Republic of China)

## Abstract

**Background:**

*Mycobacterium abscessus* is notoriously difficult to treat. Current guidelines suggest a 14-day long incubation period and/or sequencing of *erm41* to detect inducible macrolide resistance when treating pulmonary disease. However, the lengthy incubation is inconvenient, and genotyping is not routinely available nor correlated with treatment outcomes in extrapulmonary infections. We aimed to assess whether a more than two-fold change in minimum inhibitory concentrations (MICs) at D7 can reliably predict inducible macrolide resistance at D14.

**Table 1.**
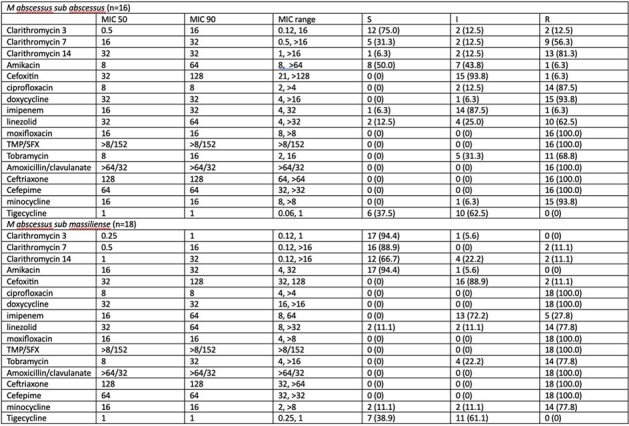
MICs of the included M. abscessus isolates against selected antibiotics and breakpoints interpretation The only M abscessus sub bolletei had macrolide MIC of 2 (S), 32 (R), 32 (R) on D3, 7, 14, and amikacin MIC of 8 (S), cefoxitin 32 (S), ciprofloxacin 8 (R), doxycycline 32 (R), imipenem 8 (I), linezolid 8 (S), moxifloxacin 8 (R), TMP/SFX > 8/152 (R), tobramycin 16 (I), amoxicillin/clavulanic acid >64/32 (R), ceftriaxone 128 (R), cefepime 64 (R), tigecycline 0.5 (S)

**Methods:**

Between 2013-2015, 35 *M. abscessus* isolates were identified from non-pulmonary sites at a medical center in Taiwan. The species were identified using mass spectrometry and sequencing of *erm41,* and *rrl* were done. Testing for MIC was done with broth microdilution, with results read on D3, D7, and D14 for macrolides, and all other antibiotics on D3. The isolate is predicted to have inducible macrolide resistance if there is a twofold or more increase in MIC between D3 and D7. The clinical information was obtained from chart review.

**Table 2.**
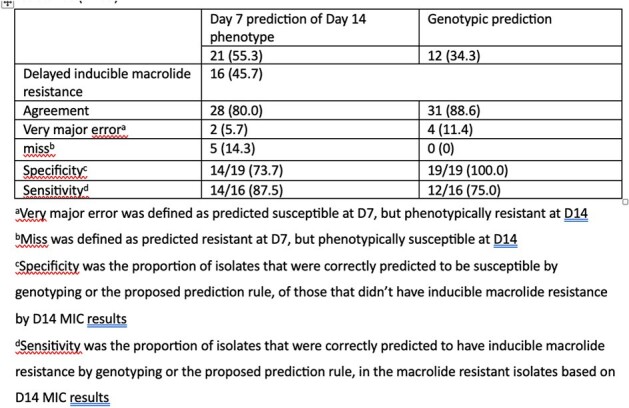
Application of macrolide prediction rule and the correlation with phenotypic and genotypic resistance (N=35)

**Results:**

There were a total of 35 unique *M. abscessus* isolates comprising 16 subsp. *abscessus*, 18 subsp. *massiliense* and 1 subsp. *bolletii*. The overall inducible macrolide resistance was 45.7% and 2 of the 5 sequevar C28 *M abscessus* exhibited inducible macrolides resistance. Using proposed prediction rule, the sensitivity and specificity was 87.5% and 73.7%, respectively; resulting in 2 very major errors (failure to identify inducible macrolide resistance), and comparatively superior to genotypic prediction, resulting in 4 very major errors. From chart review, 80% of the patients received combinatorial treatment, and macrolides were the most widely used antibiotics. 67.9% of them received surgery, or debridement. The overall clinical cure rate was 75%, after a median treatment duration of 6 months.

**Table 3.**
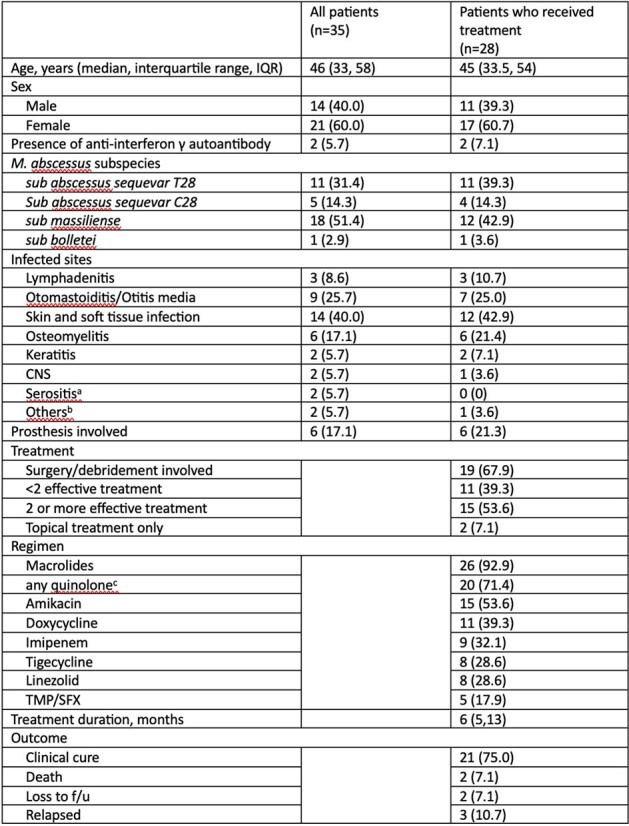
Patient characteristics a: One subject with ESKD under peritoneal dialysis had isolated culture within ascites, one man had MAB growth in pleural effusion. b: One subject had MAB growth in liver abscess, and the other had positive culture in gastric juice. c: 14 (50%) patients got moxifloxacin, 13 (46.4%) patients got levofloxacin and 5 (17.9%) got ciprofloxacin.

**Conclusion:**

The study highlighted that susceptibility testing read within one week can give meaningful information on inducible macrolide resistance that was not inferior to genotypic information. Further validation of early MIC changes to guide treatment selection is warranted.

**Disclosures:**

**All Authors**: No reported disclosures

